# Bioorganic fertilizer promotes pakchoi growth and shapes the soil microbial structure

**DOI:** 10.3389/fpls.2022.1040437

**Published:** 2022-11-08

**Authors:** Tao Wang, Keke Cheng, Xingjuan Huo, Pinpin Meng, Zhonghua Cai, Zongkang Wang, Jin Zhou

**Affiliations:** ^1^ Institute of Ocean Engineering, Shenzhen International Graduate School, Tsinghua University, Shenzhen, China; ^2^ Ecological Fertilizer Research Institute, Shenzhen Batian Ecological Engineering Co., Ltd., Shenzhen, China

**Keywords:** bioorganic fertilizer, soil microbial profiles, *Brassica chinensis* L., promote plant growth, green agriculture

## Abstract

As a functional probiotic, *Bacillus subtilis* can promote crop growth and improve nutrient utilization by various mechanisms, so it has been made into bioorganic fertilizer as a replacement for chemical fertilizer. However, the effects of *B. subtilis* bioorganic fertilizer application on the yield and quality of commercial crops of *Brassica chinensis* L., the soil physicochemical properties and the microflora have not been clarified. In this study, pot experiments were conducted using *Brassica chinensis* L. plants with four fertilization treatments: control without fertilization (CK), chemical fertilizer (CF), organic fertilizer (OF), and bioorganic fertilizer containing *B. subtilis* (BF). After 30 days of pot experiment, the results showed that BF efficiently improved plant height and biomass (1.20- and 1.93-fold, respectively); as well as significantly increasing soil available potassium and pH value. Using high-throughput sequencing, we examined the bacterial and fungal communities in the soil, and found that their diversity was remarkablely reduced in the BF treatment compared to CK group. A principal coordinate analysis also showed a clear separation of bacterial and fungal communities in the BF and CK groups. After application of *B. subtilis* bioorganic fertilizer, some beneficial bacteria (such as *Bacillus* and *Ammoniphilus*) and fungi (*Trichoderma* and *Mortierella*) were enriched. A network analysis indicated that bacteria were the dominant soil microbes and the presence of *B. subtilis* stimulated the colonization of beneficial microbial communities. In addition, predictive functional profiling demonstrated that the application of bioorganic fertilizer enhanced the function of mineral element metabolism and absorption and increased the relative abundance of saprotrophs. Overall, the application of bioorganic fertilizer effectively changed the soil microflora, improved the soil available potassium and pH value, and boosted the yield of *Brassica chinensis* L. This work has valuable implications for promoting the safe planting of facility vegetables and the sustainable development of green agriculture.

## 1 Introduction

*Brassica chinensis* L. (pakchoi) is an annual vegetable in the Cruciferous family (Yang et al., 2010). Due to its short growth cycle, high multiple cropping index and high planting efficiency, *Brassica chinensis* L. is widely cultivated (Ferreira and Ranal, 1999; Ferreira et al., 2002). However, driven by economic benefits, a high input of chemical fertilizers is employed for its cultivation. Overuse of these synthetic inputs causes adverse effects on the soil ecology and food safety, resulting in a decline of soil quality, nutrient loss and the consequences of an excessive nitrite concentration in crops (Ma et al., 2014; Cai et al., 2016). Therefore, organic, especially the bioorganic fertilizers have recently received much attention as a promising alternative strategy (Yuan et al., 2013; Ling et al., 2014).

Microorganisms, one of the most important and active parts of the soil ecosystem, play a dominant role in promoting soil nutrient cycling and maintaining system stability, and are important for the continuous functioning of soil microecological status (Emery et al., 2019; Nazaries et al., 2021). Additionally, the soil microbial community can provide essential nutrients for crop growth and stimulate crop development by various mechanisms (Yang et al., 2019b). Bioorganic fertilizer is a collection of organic fertilizers and probiotic microorganisms that can activate various microorganisms in the soil. And it is increasingly important in promoting crop production, restoring soil fertility, and inhibiting soil diseases (Huang et al., 2014; Schoebitz et al., 2014; Liu et al., 2016).

Among many bioorganic microbial fertilizers, *Bacillus subtilis*, which is widely distributed in soil and decaying organic matter, is a representative type. It has been shown in previous studies that *B. subtilis* has good regulatory effects for promoting crop growth, improving soil quality and the crop microenvironment, controlling soil-borne diseases and remediating farmland pollution (Zhao et al., 2013; Wu et al., 2016). Meanwhile, due to its excellent stress tolerance, short culture period and convenient application, *B. subtilis* has increasingly become dominant component in bioorganic fertilizer (Sun et al., 2020). Zhao et al. (2019) reported that the application of bioorganic fertilizer including *Bacillus* could reshape the soil microbial community and promote pepper growth. It was shown that the application of *Bacillus* bioorganic fertilizer could be a sustainable pathway to improve soil nutrient utilization as well as increase the yield and quality of lettuce (Jin et al., 2022). In addition, *Bacillus* bioorganic fertilizer has recently been applied in the cultivation of economic crops, and the results show that it could stimulate microbial activity, inhibit banana Fusarium wilt disease and enhance the stress resistance of sorghum (Wu et al., 2019; Wang et al., 2022). However, previous studies only investigated the effects of bioorganic fertilizers on soil physicochemical properties, crop growth and the microbial community composition, without further exploring microbial network relationships and microfloral functions. Moreover, the effects of *Bacillus* bioorganic fertilizer on the *Brassica chinensis* L. have not been well characterized.

Base on this, we hypothesized that *B*. *subtilis* bioorganic fertilizer could promote the growth of *Brassica chinensis* L. and improve soil microbial profiles (biodiversity, function and network interactons). Therefore, a novel bioorganic fertilizer was developed by fermenting mature compost with *B. subtilis* produced by the Shenzhen Batian Ecological Technique Co., Ltd; and pots experiments were performed to investigate the effects of bioorganic fertilizer on plants and soil microbial features. The specific objectives of this study were to (1) evaluate the direct effect of *B. subtilis* bioorganic fertilizer on *Brassica chinensis* L. cultivation; (2) explore the potential impact of this fertilizer on the soil characteristics; and (3) determine the alterations of soil microbial community, co-occurrence relationship and functions caused by this type of fertilizer. By revealing the mode of action of bioorganic fertilizers, this study sought to provide the necessary understanding required for the more efficient and informed development of soil microbiome manipulation strategies involving biologically enhanced organic fertilizers.

## 2 Materials and methods

### 2.1 Experimental design

Greenhouse experiments were carried out in the Shenzhen Batian Ecological Fertilizer Research Center (22° 46′ 6.13′′ N, 113° 48′ 32.70′′ E) in Shenzhen City, Guangdong Province, China from 10 December 2021 to 09 January 2022. The soil was lateritic soil with a pH of 6.30. Soil organic matter (OM), total nitrogen (TN), total soil phosphorus (TP) and potassium (TK) were 34.22, 1.83, 1.97 and 17.80 g/kg soil, respectively. Alkaline hydrolysis nitrogen (AHN), available phosphorus (AP) and available potassium (AK) were 112.22, 164.12 and 200.19 mg/kg soil, respectively. Organic fertilizer and the newly developed bioorganic fertilizer were produced by the Shenzhen Batian Ecological Technique Co., Ltd. (China). The organic fertilizer composted with chicken manure and rice husks with a 1:1 weight ratio. On the 14^th^ day, the composting was completed and granulated to make organic fertilizer containing 42.44% of OM, 1.30% of N, 0.40% of P_2_O_5_ and 4.14% of K_2_O. The bioorganic fertilizer was a secondary fermentation based on organic fertilizer. *Bacillus subtilis* AMMS-012 was added to the decomposed organic material at a rate of 5×10^10^ spores per g for further fermentation. On the 5^th^ day, granulation was carried out and air-dried at low temperature to make bioorganic fertilizer, and the number of colonies was detected as 7.5×10^8^ spores g^-1^. Commerical chemical fertilizers, including urea, monoammonium phosphate and potassium chloride purchased from Yunnan Yuntianhua Co., Ltd., China.

The soil was exposed to the sunlight for 3 days before the experiment and large particles were removed with a 2 mm sieve. The *Brassica chinensis* L. seeds were placed in the vermiculite matrix to sow and raise seedlings. After 18 days, the seedlings with consistent growth and good health were selected for transplanting. Four fertilization treatments were applied as follows: (1) BF, bioorganic fertilizer; (2) OF, organic fertilizer; (3) CF, chemical fertilizer; and (4) CK, control without fertilization. The nutrient (N, P, and K) supply among the experimental fertilization treatments was equalized by the chemical fertilizers. BF was set as the nutrient standard by applying 12,000 kg·ha^-1^ of bioorganic fertilizer to the field (Kang et al., 2022). Specifically, BF was applied with 5.0 g bioorganic fertilizer per kilogram of soil in the pots, and the nutrient supply of the other treatments was made equal to it. Bioorganic and organic fertilizers were applied as the base fertilizer once; and for the chemical fertilizer, one-third of the total amount of chemical fertilizer was applied as a basal fertilizer. The remainder of the chemical fertilizer was applied 10 or 20 days after the seedlings were planted. Six pots (replicates) were set up for each treatment, and each pot contained 4 kg soil and three *Brassica chinensis* L. seedlings. The pots were randomly arranged in the greenhouse with the temperature maintained at 25 ± 1°C under natural light. Conventional operations such as watering, scarifying and disinsection were applied equally when needed. Plants were grown for 30 days and the growth parameters (morphological characteristics) were measured every 5 days, and plant and soil samples were collected on the 30^th^ day for quality and diversity index determination.

### 2.2 Soil physicochemical analysis

The soil pH value was determined with a glass electrode using a soil-to-water ratio of 1:2.5 (w/v). OM was determined by a K_2_Cr_2_O_7_ oxidation-reduction titration method and the Kjeldahl method was used for TN estimation (Guimaraes et al., 2013). TP and TK were digested by HF-HClO_4_ and determined by molybdenum-blue colorimetry and flame photometry, respectively (Shen et al., 2008). AHN in soil was determined based on the transformation of hydrolyzed nitrogen into ammonia nitrogen with sodium hydroxide (Li et al., 2013). AP in soil was extracted with sodium bicarbonate and determined using the molybdenum blue method (Hedley et al., 1982). AK in soil was extracted with ammonium acetate and determined with inductively coupled plasma spectrophotometry (ICP-9000, Shimadzu, Japan) (Fukuda et al., 2017).

### 2.3 Plant trait analysis

Thirty-six *Brassica chinensis* L. plants were randomly selected from each treatment plot, and the height, crown width and leaf width of the plants were measured with a tape measure (Liu et al., 2014; Amoah et al., 2017). A handheld, non-destructive SPAD chlorophyll meter (SPAD-502, Konica Minolta, Japan) was used to measure the chlorophyll concentration in the plant leaves (Li et al., 2022c). The leaves of each plant were numbered from the bottom to the top of the stem (Alemayehu et al., 2020). Plant samples (shoot and root) were washed and laid on paper towels to dry, and the fresh weight was recorded. Plant samples were subsequently dried at 70°C for 120 hours before the dry weight was recorded (Cai et al., 2015).

The soluble sugar concentration was measured by anthrone colorimetry (Ibrahim et al., 2013). The soluble protein concentration was determined spectrophotometrically by measuring the absorbance at 595 nm with bovine serum albumin as the standard (Bradford, 1976). The nitrate determination used the salicylic acid colorimetric method (Cataldo et al., 1975).

### 2.4 Soil DNA extraction, PCR amplification and high-throughput gene sequencing analysis

DNA from all samples was extracted and purified (the details are described in the Additional file). Two specific primers were used to amplify the bacterial 16S rRNA gene and the fungal ITS1 region (Schoch et al., 2012; Mori et al., 2014). Sequencing was performed on an Illumina Miseq platform (Majorbio Bio-Pharm Technology Co., Ltd., Shanghai, China). Sequences were comparedagainst the Silva (SSU123) 16S rRNA Database and the fungal (ITS) Unite Database to obtain the species annotation information (Li et al., 2022c). Sequence data associated with this project have been deposited in the NCBI Short Read Archive database (Accession Numbers: PRJNA862974 and PRJNA863750).

### 2.5 Network analysis

Network analysis was performed based on OTUs to explore the relationships between the soil microbial taxa as described in Additional file. Data set was calculated by Spearman correlation matrix and adjusted for Benjamini-Hochberg’s false discovery rate, and thenetwork visualization as well as topological parameters were analyzed using Gephi 0.9.5 (https://gephi.org/) (Benjamini and Hochberg, 1995; Li et al., 2019).

### 2.6 Statistical analysis

Statistical analysis of the soil physicochemical properties and plant agronomic traits was carried out by applying a one-way analysis of variance (ANOVA) and the new multiple range method. All experimental parameters were measured at least in triplicate, and the results were expressed as the mean ± standard deviation. Difference analyses were conducted with the SPSS statistical software package, version 22.0 (IBM, New York, USA), with a *P* value ≤ 0.05 as the standard.

To compare the relative levels of OTU diversity across all samples, a rarefaction curve was formed using the Mothur software (Schloss et al., 2009). Alpha diversity, including the Chao 1 and Shannon indexes, were calculated using QIIME (version 1.9.1) (Zhang et al., 2022). The relative abundance of microbial phylum and genus were defined as the number of reads of that phylum and genus as a percentage of the number of all reads in a sample (Yao et al., 2019). To compare bacterial and fungal community structures among all soil samples, a principal coordinate analysis (PCoA) was set up based on the Bray–Curtis distance metric (Yang et al., 2022). Theigraph and psych packages in R (version 4.2.0) were used to visualize the results (Kang et al., 2022). An ANOVA was used to determine the statistical significance of the differences between species and treatment groups, and the FDR (False Discovery Rate) multiple test was used for correction. A linear discriminant analysis (LDA) effect size (LEfSe) method was performed to find biomarkers (LDA score ≥ 3.5) (Li et al., 2022c).

The function of bacterial communities was predicted using the PICRUSt software program according to the Kyoto Encyclopedia of Gene and Genomes (KEGG) catalog (Langille et al., 2013), and the function of fungal communities was predicted using the FUNGuild software (Nguyen et al., 2016). The results were graphed primarily using Origin 2022 (OriginLab, USA) and Adobe Illustrator CC2018 (Adobe Systems Inc., USA) (Li et al., 2022c). In addition, a structural equation models (SEMs) analysis was conducted by SPSSAU using an online tool (https://spssau.com) (Li et al., 2022a).

## 3 Results

### 3.1 Agronomic traits of *Brassica chinensis* L.

#### 3.1.1 Plant biomass and growth factors

The overall trend for plant biomass was that fertilizer treatments were significantly higher than CK, especially BF (**Table S1**, **Figure S1**). During the experimental period, the shoot fresh weight, root fresh weight, total fresh weight, and total dry weight were 83.03 ± 10.89, 2.27 ± 0.29, 85.29 ± 11.07, and 4.26 ± 0.43 (g/plant), respectively; which is 1.81–1.93-fold higher than CK (*P* ≤ 0.05). It was noteworthy that no significant difference was evident between CF and OF, but both were remarkablely lower than the BF group.

For other plant growth factors, compared with CK, all treatments showed greatly increased plant height, crown width and the third leaf width, especially BF (**Table S2**, **Figure S1**). Compared with the other three treatments, BF showed dramatically increased plant height, by 5.43%–20.00% on the 30^th^ day. For the leaf number, significant differences were seen among the three test groups and CK in the early stages (i.e., 5 d and 10 d), but the difference in the three test groups was not obvious. From 15 d to 30 d, the leaf number in BF group increased significantly compared with all other groups (CF, OF and CK) (**Table S3**). Unlike the leaf number, the leaf SPAD value was not statistically different among BF and the other groups in the early stages. A significant difference was observed at 30 d (**Table S3**).

#### 3.1.2 Quality characteristics

**Figure 1:**
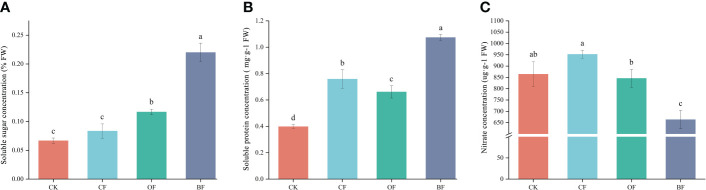
Effects of different fertilizer treatments on the *Brassica chinensis* L. soluble sugar concentration **(A)**, soluble protein concentration **(B)** and nitrate concentration **(C)**. CK is the untreated control; CF is chemical fertilizer; OF is organic fertilizer; BF is bioorganic fertilizer; FW is fresh weight. Means (N = 3) within the same histogram followed by the same letter are not statistically different (*P* = 0.05) according to Duncan’s new Multiple-Range test. The different small lettles (a, b, c, and d) indicated the significant difference among the different groups at P<0.05 level.

The soluble sugar concentration of BF and OF was 0.22% and 0.12%, respectively, which was significantly higher than CK (**Figure 1A**). CK showed the lowest soluble protein concentration, whereas BF was increased by 40.79% and 62.12% compared with CF and OF, respectively (**Figure 1B**). Unlike the soluble sugar/protein, the nitrate concentration showed a different tendency. BF showed the lowest nitrate with a 30.34% and 23.27% decrease compared with CF and CK, respectively (**Figure 1C**). These results indicated that application of organic fertilizer effectively improved the quality of *Brassica chinensis* L.

### 3.2 The relationship between plant properties and environmental factors

Compared with CK, all treatments except CF showed significantly increased alkaline hydrolysis nitrogen (AHN), available potassium (AK) and organic matter (OM). Especially in, BF group, which effectively increased the AK concentration by 62.21% (**Table S4**). Additionally, available phosphorous (AP) was dramatically increased (8.69%–14.87%) after fertilization, and the largest increase occurred in BF. At the same time, both BF and OF also had improved soil acid-base status indicated by a higher pH value. The pH value of CK was 6.22 ± 0.01, whereas the value was 6.60 ± 0.08 in BF.

A correlation analysis showed that plant weight, the leaf SPAD value, and the soluble sugar and protein concentration were positively correlated with the environmental factors, whereas the nitrate concentration was negatively correlated with the environmental factors (**Table S5**). Plant fresh weight was positively correlated with AHN, AP, AK and OM (*P* ≤ 0.05), and SPAD was positively correlated with AP, AK and pH (*P* ≤ 0.05). The nitrate level was negatively correlated with AK, OM and pH (*P* ≤ 0.05). The soluble sugar concentration was positively correlated with all environmental factors values (*P* ≤ 0.01); and the soluble protein concentration was positively correlated with AHN, AP and AK concentrations (*P* ≤ 0.05).

### 3.3 Soil microbial diversity

#### 3.3.1 Biodiversity of the soil microbial community

After optimizing the original sequence based on a 97% similarity, 37,014 16S rRNA and 58,153 ITS sequences were retained from all samples; a total of 4,673 bacterial OTUs and 1,549 fungal OTUs were obtained. The Shannon and Chao1 indices respectively represent the diversity and richness of microbial communities.

Compared with BF, more OTUs were observed in CF and CK for bacteria and fungi, and CK had the highest value of all treatments (**Table S6**). In BF, the Chao1 richness of bacteria decreased by 3.21% and 7.01% compared with CF and CK. However, in the fungal biosphere, no significant differences in the Chao1 index occurred among the four treatments. For the Shannon index, CK showed the highest value for both bacteria and fungi. Overall, CK and CF had higher microbial α-diversity compared with BF and OF.

**Figure 2:**
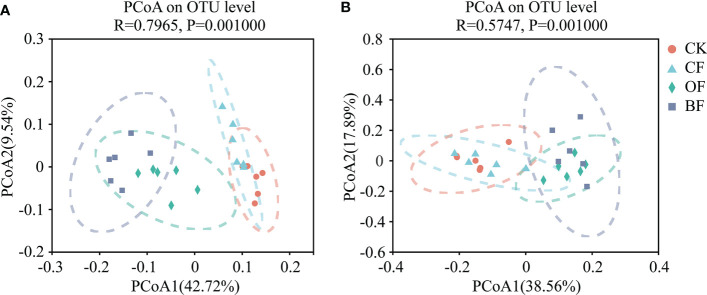
Comparison of the bacterial **(A)** and fungal **(B)** communities in soils under the different fertilizer treatments based on a principal coordinates analysis of Bray-Curtis distances. CK is untreated control; CF is chemical fertilizer; OF is organic fertilizer; BF is bioorganic fertilizer.

For β-diversity, The differences in community composition of the four treatments were evaluated by using a PCoA based on a Bray-Curtis distance matrix. **Figure 2** shows that application of organic fertilizer accounts for the differences in the composition of the soil microbial community. Both bacterial and fungal cluster into two distinct groups representing samples taken from four treatments. The Bray–Curtis distances show that BF was separate from CF and CK along the first component (PCoA1) both for bacteria and fungi (PERMANOVA, F = 6.6612, *P* = 0.001, bacteria; F = 5.30167, *P* = 0.001, fungi). The contribution rates of PCoA1 and PCoA2 to the differences in species composition between the treatments were 42.72% and 9.54% (bacteria), and 38.56%, 17.89% (fungi), respectively.

#### 3.3.2 Soil microbial community composition

**Figure 3:**
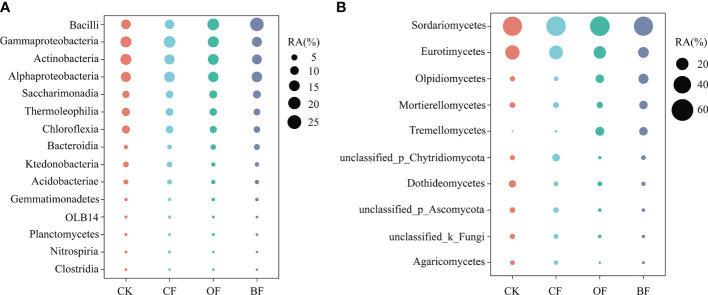
Bubble chart of bacterial **(A)** and fungal **(B)** classes for different fertilizer treatments. CK is untreated control; CF is chemical fertilizer; OF is organic fertilizer; BF is bioorganic fertilizer. The circular areas represent the average relative abundance (RA) across the six replicate libraries for soil samples collected from each treatment.

For the bacteria, Proteobacteria, Actinobacteria, Firmicutes, Chloroflexi and Patescibacteria were the top five abundant phyla (**Figure S2A**). At the class level, Bacilli, Gammaproteobacteria, Actinobacteria, Alphaproteobacteria, Saccharimonadia, Thermoleophilia and Chloroflexia are the main species (**Figure 3A**). For the fungi, Ascomycota, followed by Olpidiomycota, Basidiomycota, Mortierellomycota and Chytridiomycota were the abundant phyla (**Figure S2B**). The class level was dominated by Sordariomycetes, Eurotiomycetes, Olpidiomycetes, Mortierellomycetes and Tremellomycetes (**Figure 3B**).

**Figure 4:**
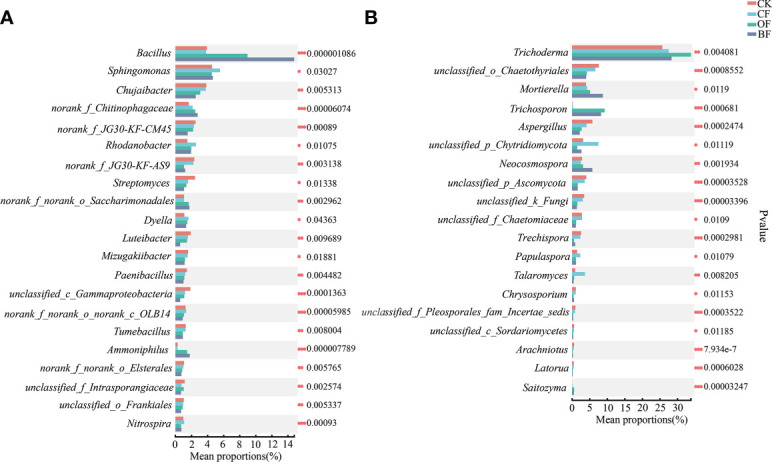
Differences in the composition of soil bacteria **(A)** and fungi **(B)** at the genus level in response to different fertilizer treatments. CK is untreated control; CF is chemical fertilizer; OF is organic fertilizer; BF is bioorganic fertilizer. The number of asterisks indicates significant differences between treatments according to a one-way ANOVA and FDR (False Discovery Rate) adjustment: * 0.01 < *P* ≤ 0.05; ** 0.001 < *P* ≤ 0.01; *** *P* ≤ 0.001.

At the genus level, *Bacillus* was the dominant genus in the bacterial community, both OF and BF dramatically (*P* ≤ 0.001) increased the relative abundance of *Bacillus* by 129.18% and 278.01% (**Figure 4A**). Addition of bioorganic fertilizer in BF also significantly increased the relative abundance of *Ammoniphilus* and norank_f_Chitinophagaceae. Among the declining species, BF significantly decreased (*P* ≤ 0.01) the relative abundance of *Chujaibacter* and *Streptomyces* by 34.53% and 56.65%, respectively.

*Trichoderma* was the dominant genus in the fungal community. All treatments increased the relative abundance of *Trichoderma* by 7.13%–31.84% (**Figure 4B**); while significantly reduced the relative abundance of *Aspergillus*. The addition of bioorganic fertilizer in BF group greatly enriched the relative abundance of *Mortierella*, *Trichosporon* and *Neocosmospora*, and decreased the relative abundance of *Trechispora*.

A LefSe analysis showed that at the bacterial genus level, 6, 2, 2 and 4 biomarkers were found in CK, CF, OF and BF, respectively (**Figure S3A**). At the fungal genus level, 8, 4, 2 and 3 biomarkers were found in CK, CF, OF and BF, respectively (**Figure S3B**).

### 3.4 Relationship among microbial community, environmental factors and agronomic traits

**Figure 5:**
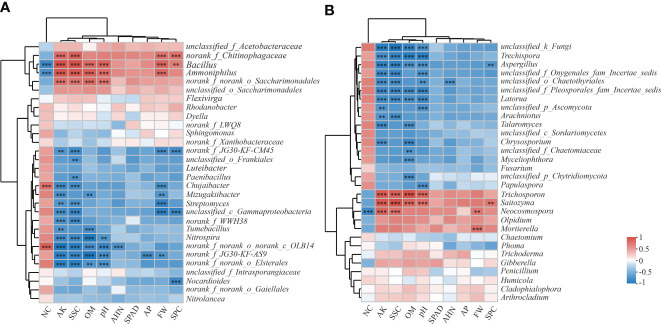
Heatmap analysis of the correlation between the species composition of soil bacteria **(A)** and fungi **(B)** at the genus level and environmental factors and agronomic traits. CK is untreated control; CF is chemical fertilizer; OF is organic fertilizer; BF is bioorganic fertilizer; FW is fresh weight; SPAD is leaf SPAD value; NC is nitrate concentration; SSC is soluble sugar concentration; SPC is soluble protein concentration; AHN is alkaline hydrolysis nitrogen; AP is available phosphorus; AK is available potassium; OM is organic matter. The Spearman method was used for correlation analysis. The legend on the right is the color interval for the different R values. The number of asterisks indicates the degree of correlation: *0.01 < *P* ≤ 0.05; **0.001 < *P* ≤ 0.01; *** *P* ≤ 0.001.

The correlation analysis at the genus level showed that bacteria were more closely correlated with the soil parameters and agronomic traits than the fungi were (**Figure 5**). In the bacterial community, *Bacillus* and *Ammoniphilus* were positively correlated with AK, OM, pH, FW, SSC and SPC (*P* ≤ 0.01); whereas negatively correlated with NC (*P* ≤ 0.001) (**Figure 5A**). *Chujaibacter*, *Mizugakiibacter* and *Streptomyces* were negatively correlated with FW and AK (*P* ≤ 0.01). *Paenibacillus*, *Streptomyces* and *Nitrospira* were negatively correlated with SSC (*P* ≤ 0.01) and *Mizugakiibacter*, *Tumebacillus* and *Nitrospira* were negatively correlated with OM (*P* ≤ 0.01).

In the fungal community, *Trechispora*, *Aspergillus* and *Latorua* were negatively correlated with AK, OM, pH and SSC; whereas *Trichosporon* and *Saitozyma* were positively correlated with these factors (*P* ≤ 0.001) (**Figure 5B**). *Neocosmospora* was positively correlated with AK, FW and SSC (*P* ≤ 0.01); and negatively correlated with NC (*P* ≤ 0.001). *Talaromyces* and *Chrysosporium* were negatively correlated with AK and OM (*P* ≤ 0.001).

### 3.5 Network analysis and predictive functional profiling of the soil microbial community

**Figure 6:**
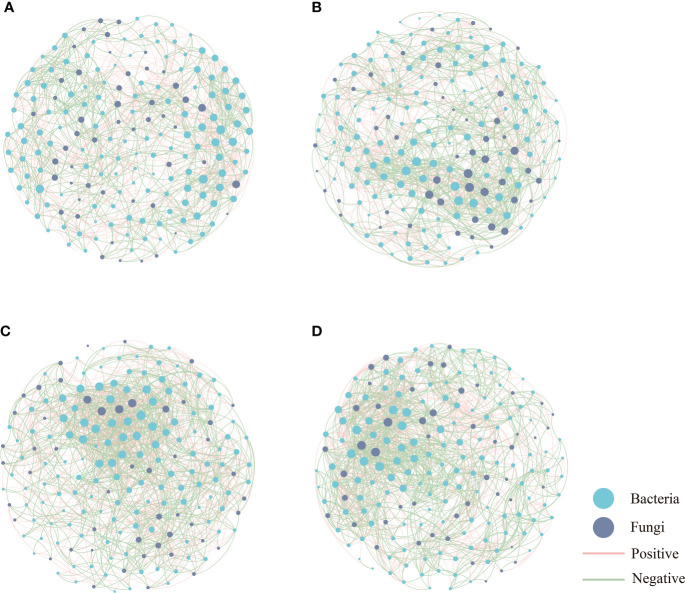
Network plots of bacterial and fungal communities in CK **(A)**, OF **(B)**, CF **(C)** and BF **(D)**, CK is untreated control; CF is chemical fertilizer; OF is organic fertilizer; BF is bioorganic fertilizer. Blue nodes indicate bacteria; gray nodes indicate fungi; red lines between nodes indicate positive interaction; and green lines indicate negative interaction.

The co-occurrence networks of microbial communities were constructed for bacteria and fungi to show interactions among genera based on strong and significant correlations (Spearman’s correlation coefficient |r| > 0.6, *P* < 0.05, FDR-BH tests) (**Figure 6**). Topological features of the global co-occurrence networks are listed in **Table S7**. A network analysis showed that the co-occurrence network was more complex in BF compared to the other groups, which consisted of 189 nodes and 1541 edges. The proportion of positive correlation accounts for 50.75%–57.82% in the four networks. The addition of organic amendments (i.e., in OF and BF) increased the network density and the average degree of the microbial networks. Additionally, the average path length decreased from 3.093 to 2.936 following the bioorganic fertilization of the soil.

Regarding microbial function, compared with the other three treatments, microbial activity in sulfur metabolism, phosphonate and phosphinate metabolism were significantly higher in BF (*P* ≤ 0.01), while other metabolic pathways, such as biofilm formation and plant-pathogen interaction were down-regulated (**Figure S4**). Significant differences were also seen in carbon fixation, mineral absorption and metabolism among the four treatments. Seven trophic modes of fungi were identified among the different fertilization treatments. They were saprotroph (51.14%–64.45%), pathotroph-saprotroph-symbiotroph (8.97%–15.35%), pathotroph (5.20%–8.21%), saprotroph-symbiotroph (4.00%–8.75%), pathotroph-saprotroph (1.71%–3.29%), symbiotroph (0.02%–0.06%), pathotroph-symbiotroph (0.02%–0.04%) and unassigned (10.88%–20.53%), respectively. (**Figure S5A**). Saprotroph was the dominant trophic mode and the value for BF was larger than for the other treatments. In addition, the pathotroph mode of BF was significantly lower compared with CK. The dominant groups of functional soil fungi were undefined saprotroph, endophyte-litter saprotroph-soil saprotroph-undefined Saprotroph, animal pathogen-dung saprotroph-endophyte-epiphyte-plant saprotroph-wood saprotroph and wood saprotroph, and the average abundance of all treatments accounted for 49.95%, 7.21%, 5.94% and 4.61%, respectively (**Figure S5B**). The abundance of unclassified saprotroph in BF increased by 19.54% compared with CK, and the abundance of animal pathogen-dung saprotroph-endophyte-epiphyte-plant saprotroph-wood saprotroph decreased by 35.71%. In addition, BF showed a significantly increased abundance of wood saprotroph over the other treatments.

### 3.6 Linkage of microbial andenvironmental factors to plant growth

**Figure 7:**
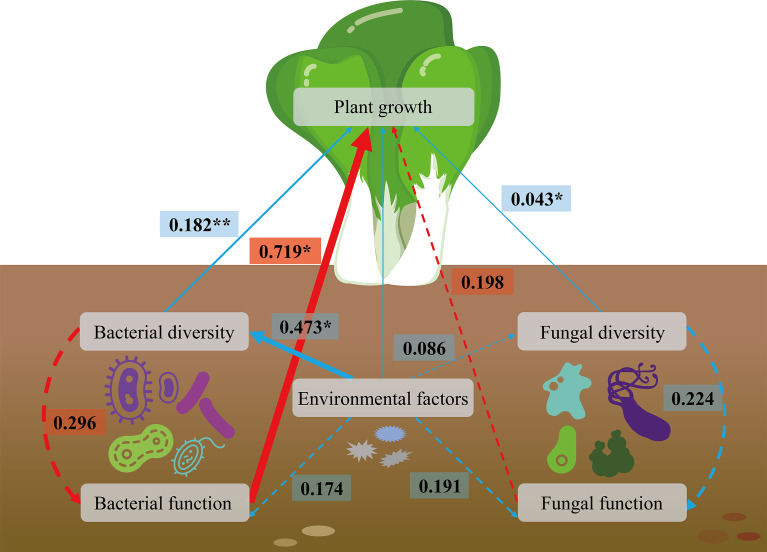
Structural equation modeling (SEM) showing the linkage among environmental factors, species diversity, species function, and plant growth. Dotted arrows indicate non-significant paths (*P* > 0.05). Red and blue arrows indicate positive and negative relationships, respectively. The path widths are scaled proportionally to the path coefficient. *0.01 < *P* ≤ 0.05, **P < 0.01.

The effects of microbial diversity, microbial function and environmental factors on plant development were evaluated using the structural equation model (SEM). The findings indicated that the plant growth was positively co-associated with the bacterial diversity and fungal diversity (**Figure 7**). Both the environmental factors and microbial diversity positively correlated with the microbial function; and the microbial function was promoted plant growth. Moreover, the environmental factors enhanced bacterial diversity, which improved the growth of *Brassica chinensis* L., indirectly. Taken together, in the analyzed parameters, plant growth was co-regulated bymicrobial composition, microbial metabolic potential and environmental factors. Among them, bacterial function, bacterial diversity, and fungal diversity showed most obvious effects on the growth of *Brassica chinensis* L. with R^2^ values ranging from 0.043 to 0.719, and *P* ≤ 0.05 or 0.01. These results showing that soil microbial profile was an important factor affecting crop growth.

## 4 Discussion

This study showed that soil physicochemical properties changed significantly after bioorganic fertilizer application. Specifically, both pH value and AK level were significantly improved in BF group compared with the control group (**Table S4**). Previous studies revealed that increase in soil pH index may be ascribed to the decomposition of organic matter, and the application of bioorganic fertilizer introduced a large amount of organic matter and microorganisms, thus the decomposition of organic matter can be accelerated by microbes to alleviate soil acidification (Hu et al., 2018; Yang et al., 2019a). The efficient soil microorganisms influence the availability of minerals in soil and play a major role in ion cycling and soil fertility. Abou-el-Seoud and Abdel-Megeed (2012) used *B. subtilis* with *B. mucilaginosus* to develop efficient microbial consortium, which helps to enhance the potassium availability in agricultural soils. In addition, many researchers have reported that a wide range of rhizosphere microorganisms can act as K solubilizers, including *B. circulans*, *B. edaphicus* and *B. amyloliquefaciens* (Sheng, 2005; El-Hadad et al., 2011). They solubilize the insoluble potassium to soluble forms of potassium mainly by chelation, exchange reactions, complexolysis, and production of organic acids. In this work, the correlation analysis shows that soil AHN, AP, AK, OM and pH were all characteristic factors affecting *Brassica chinensis* L. growth. Among these parameters, AK has the highest factor loadings and the biggest contribution for plant growth (**Tables S1, S2, 3**, **Figure S1**). Hency, our results show that bioorganic fertilizer increased the availability of potassium and provides necessary element for plant growth. Meanwhile, this study also revealed that the addition of *B. subtilis* bioorganic fertilizer significantly reduced the nitrate concentration in the crop, probably because the soil microbial amendment reduced the nitrate concentration by increasing the nitrate reductase activity, thereby improving the safety and quality of vegetables (Chatterjee et al., 2012; Harindintwali et al., 2021). Previous studies have confirmed that additional bacterial fertilizers can reduce nitrate concentration in vegetables, such as lettuce and tomato (Brunetti et al., 2019; Jiang et al., 2019; Jin et al., 2022). Jin et al. (2022) pointed out the application of *B. subtilis* bioorganic fertilizer enriched organic matter concentration in the plant-body, which increased the quality of lettuce. Similar results were observed in the present study, where soluble sugar and protein levels were increased to varying degrees in the BF group. This may be related to the effective promotion of nutrient metabolism coordination and the balance of organic fertilizer, so as to ensure a higher yield and better quality of crops (Brunetti et al., 2019).

Base on plant growth or quality is closely related to soil fertility and microecological characteristics (Wu et al., 2021), an analysis of the soil microbial community of *Brassica chinensis* L. was performed. For the biodiversity, it was found that *B. subtilis* bioorganic fertilizer could significantly change rhizosphere microbial diversity. Both the diversity and richness of bacteria and fungi were significantly lower for BF compared to CK (**Table S6**). A possible reason is that the bacterial fertilizer changed the composition of the soil organic matter and enriched the relatively dominant species. Previously, Jiang et al. (2019) pointed out that bioorganic fertilizer is rich in organic components and exogenous strains that can modify the habitat of soil microorganisms. Yang et al. (2022) subsequently showed that the *B. subtilis* is a typical plant growth-promoting rhizobacteria (PGPR), which can dominantly colonize the soil, decreasing the biodiversity of soil species. At the same time, Wang et al. (2017) pointed out that bioorganic fertilizer is a source of organic matter which promotes the growth of native microbes in soil and decrease the biodiversity. The above-mentioned studies provide evidence for our explanation.

For the microbial communities, the phylum level results showed that the abundance of Firmicutes was higher and Acidobacteriota was lower after the bioorganic fertilizer addition. Previous study reported that Firmicutes are k-strategy species often found in nutrient-rich habitats and can produce antibacterial substances against harmful bacteria and promote host growth (Li et al., 2022c). Unlike the Firmicutes, Acidobacteriota are an r-strategy species and prefer to inhabit an oligotrophic environment, so they show a relatively higher abundance in CK (Liang et al., 2020). In the fungi, compared with the control, bioorganic fertilizer application increased the abundance of Basidiomycota and Mortierellomycota, both of which are key fungal members engaging in nutrient conversion and material decomposition (Detheridge et al., 2016; Kumar et al., 2022). In addition, the PCoA analysis revealed that significant clustering in bacterial and fungal communities in the various treatment groups, which indicated that the bioorganic fertilizer greatly affected the microbial community (Wang et al., 2022).

At the genus level, as expected, it was found that *Bacillus* was highly enriched in the bioorganic fertilizer treatments. And the Spearman correlation test showed that the relative abundance of *Bacillus* exhibited a strong positive correlation with soil environmental factors and plant physiological parameters, which might be related to the facility of *Bacillus* for humification and the mineralization of nutrients (such as C and N), the inhibition of pathogens, and the induction of host resistance (Wu et al., 2019; Lebedev et al., 2021; Li et al., 2022c). Similar to *Bacillus*, it was also observed that norank_f_Chitinophagaceae was remarkably increased in BF compared with CK. This species degrades chitin and cellulose by secreting β-glucosidase, and inhibits harmful fungi and promotes plant growth (Xu et al., 2021). In this study, the application of bioorganic fertilizer effectively enhanced the yield of *Brassica chinensis* L., which may be related to the high relatively abundance of norank_f_Chitinophagaceae. Furthermore, *Ammoniphilus* was significantly enriched in the organic fertilizer treatments and had a strong positive correlation with crop physiological indicators and soil nutrient parameters, probably related to its ability to secrete organic matter hydrolases to accelerate substance degradation and promote nutrient element metabolism (Otto et al., 2013).

In the fungal biosphere, the *Trichoderma*, *Mortierella*, *Trichosporon* and *Saitozyma* were greatly increased in BF. *Trichoderma* is a typical activity member, which colonizes the rhizosphere of crops and increases the surface area of the root system to improve the nutrient absorption capacity of crops (Cai et al., 2017). Additionally, it has been found that *Trichoderma* inhibits the growth of other pathogenic microorganisms by secreting antibiotics and hydrolases, and releases slow-effect nutrients in the soil (Zhang et al., 2016). In the present study, the organic fertilizer treatment groups BF and OF had a high soil nutrient concentration and excellent crop growth parameters, which may be related to the significant enrichment of the abundance of *Trichoderma*. Similarly, *Mortierella* was significantly enriched in the organic fertilizer treatment and positively correlated with crop fresh weight, probably related to its ability to improve nutrient availability and protect crops from pathogens (Ozimek and Hanaka, 2021).

As for *Trichosporon* and *Saitozyma*, the relative abundance of both was increased in the organic/bioorganic fertilizer groups, and was significantly positively correlated with the soil organic matter, available potassium, and appropriate pH, which may be related to their ability to accelerate cellulose degradation (Stursova et al., 2012; Aliyu et al., 2021). It is also noteworthy that the abundance of *Aspergillus* decreased significantly after the application of bioorganic fertilizers, and the correlation test results showed that *Aspergillus* was negatively correlated with crop quality indicators. This indicates the plants are less likely to be damaged by toxins after applying bioorganic fertilizers, because *Aspergillus* is a common toxin producer fungus often infecting cash crops such as peanuts and corn (Senghor et al., 2020). The above-mentioned results imply that the promotion of beneficial fungi and the inhibition of harmful fungi by organic fertilizers promote plant growth.

The co-occurence analysis indicated the bacteria nodes occupy three-quarters of the network, with a higher proportion of positive than negative correlations, implying bacterial dominance in the microflora and species niches dominated by similarity (Zhang et al., 2022). In this study, the network topological parameters, including number of nodes and edges, the average degree, the network density and the average clustering coefficient of BF were much greater than the other groups, indicating that BF evidenced a higher degree of cooperation and communication (Li et al., 2022a). In addition, the high connectivity implies a great network complexity, which is important for the ecological buffer capability and microbial homeostasis (Kang et al., 2022). It was observed that the complexity of the co-occurrence networks of bacteria and fungi in BF was increased, consistent with previous studies (Gu et al., 2019; Price et al., 2021). The results of our study showed that BF showed a shorter average network path length, suggesting that the application of bioorganic fertilizer improved the efficiency of the co-occurrence network to quickly respond to environmental changes (Kang et al., 2021; Zhu et al., 2022).

For the functional profiles of the microflora, the soil bacterial and fungal ecological functions of the different treatment groups of *Brassica chinensis* L. were predicted by the PICRUSt2 and FUNGuild methods, respectively. The KEGG results showed that exposure of *B. subtilis* bioorganic fertilizer significantly enhanced the metabolism and absorption pathways of mineral elements and sulfur/phosphonate/phosphinate metabolism, which was mainly because the addition of *B. subtilis* stimulated the proliferation of other beneficial microorganisms (including bacteria and fungi). Previous studies also found that the addition of organic fertilizers significantly improved the material metabolism pathway and played an important role in nutrient cycling or decomposition, which was consistent with the predictions of this study (Cai et al., 2017; Liao et al., 2019; Li et al., 2022b). Su et al. (2022) further pointed out that organic fertilizer application improved the soil structure and stimulates beneficial microorganisms, reducing the relative abundance of pathogenic fungi. However, it should be noted that the present results are based on prediction tools, and the actual molecular functions have yet to be confirmed by subsequent genomics or transcriptomics in the future. At the same time, genetic engineering and genome editing for improving nutrients provide efficiency in microbes are needs to be strengthened in the future.

## 5 Conclusion

The present study revealed that *B. subtilis* bioorganic fertilizer can maintain the acid base balance of soil and effectively improve the plant nutritional quality by regulate the pH value and soluble matters content (sugar, protein and nitrate). Meanwhile, the soil microorganisms profiles were improved under the bioorganic fertilizer. The biofertilizer decreased the α diversity and increased soil beneficial microorganisms proliferation. Network analyses revealed closely correlations between bacteria/fungi and soil environmental parameters and crop agronomic traits. Functional profiling revealed that the application of biofertilizer promoted an increase of microbial functional pathways, such as mineral element metabolism and saprotrophy. The SEM results further supplied the evidence that crop growth was co-controlled by the microbial communities and related functions. As a whole, bioorganic fertilizers can effectively improve soil available potassium and pH value as well as microbial activities, and promote *B. chinensis* L. growth. This study provides a scientific basis for establishing an environmental friendly fertilization technology for green agriculture.

## Data availability statement

The datasets presented in this study can be found in online repositories. The names of the repository/repositories and accession number(s) can be found in the article/**Supplementary Material**.

## Author contributions

TW: writing-original draft preparation, software, visualization, writing-reviewing and editing; KC: conceptualization, writing-review and process suggestion; XH: project administration, investigation and supervision; PM: supervision and material assistance; ZC: formal analysis and methodology; ZW: experimental design, guidance and funding acquisition; JZ: writing-reviewing and editing, project administration, and funding acquisition. In addition, all authors contributed to the article and approved the submitted version.

## Funding

This work was supported by the NSFC (41976126), the S&T Projects of Shenzhen Science and Technology Innovation Committee (KCXFZ20201221173211033, RCJC20200714114433069, JCYJ20200109142818589 and WDZC20200819173345002), as well as the Project of Shenzhen Municipal Bureau of Planning and Natural Resources (Grant No. [2021]735-927).

## Acknowledgments

We are grateful to Shenzhen Batian Ecological Fertilizer Research Center for providing the experimental assistance.

## Conflict of interest

Authors XH, PM and ZW are employed by Shenzhen Batian Ecological Engineering Co., Ltd.

The remaining authors declare that the research was conducted in the absence of any commercial or financial relationships that could be construed as a potential conflict of interest.

## Publisher’s note

All claims expressed in this article are solely those of the authors and do not necessarily represent those of their affiliated organizations, or those of the publisher, the editors and the reviewers. Any product that may be evaluated in this article, or claim that may be made by its manufacturer, is not guaranteed or endorsed by the publisher.
