# Supplementary material for: Bioorganic fertilizer promotes pakchoi growth and shapes the soil microbial structure
Source: Front Plant Sci. 2022 Nov 8;13:1040437. doi: 10.3389/fpls.2022.1040437 (PMC9679507; doi:10.3389/fpls.2022.1040437)
Supplement: Supplementary file 1 [file DataSheet_1.docx]

Supplementary Material

# Supplementary Figures and Tables

## Supplementary Tables

**Supplementary Table 1****.** Effects of different fertilizer treatments on *Brassica chinensis* L. biomass.

| **Treatment** | **Shoot fresh weight (g/plant)** | **Root fresh weight (g/plant)** | **Total fresh weight (g/plant)** | **Total dry weight (g/plant)** |
| --- | --- | --- | --- | --- |
| CK | 42.92 ± 4.92^c^ | 1.25 ± 0.13^c^ | 44.17 ± 4.99^c^ | 2.31 ± 0.42^c^ |
| CF | 61.21 ± 5.98^b^ | 1.76 ± 0.27^b^ | 62.96 ± 6.12^b^ | 3.05 ± 0.34^b^ |
| OF | 63.06 ± 11.33^b^ | 1.53 ± 0.41^bc^ | 64.59 ± 11.70^b^ | 3.38 ± 0.62^b^ |
| BF | 83.03 ± 10.89^a^ | 2.27 ± 0.29^a^ | 85.29 ± 11.07^a^ | 4.26 ± 0.43^a^ |

CK = untreated control; CF = chemical fertilizer; OF = organic fertilizer; BF = bioorganic fertilizer. Means (N = 6) within the same column followed by the same letter are not statistically different (P = 0.05) according to Duncan’s new Multiple-Range test.

**Supplementary Table 2.** Effects of different fertilizer treatments on *Brassica chinensis* L. plant growth.

| Treatment | Time | | | | | | | | | | |
| --- | --- | --- | --- | --- | --- | --- | --- | --- | --- | --- | --- |
|  | 5d | | | | 10d | | | | 15d | | |
|  | Plant height/cm | Crown width/cm | Leaf width/cm |  | Plant height/cm | Crown width/cm | Leaf width/cm |  | Plant height/cm | Crown width/cm | Leaf width/cm |
| CK | 2.82±0.15^d^ | 4.74±0.62^c^ | 1.22±0.15^d^ |  | 3.64±0.22^c^ | 7.42±0.48^d^ | 2.07±0.19^c^ |  | 6.36±0.52^d^ | 9.20±0.67^d^ | 2.58±0.31^c^ |
| CF | 3.23±0.24^c^ | 6.56±0.52^b^ | 1.82±0.20^c^ |  | 4.10±0.28^b^ | 8.83±0.37^c^ | 2.56±0.19^b^ |  | 7.01±0.55^c^ | 11.59±0.68^c^ | 3.36±0.22^b^ |
| OF | 3.48±0.22^b^ | 7.06±0.74^ab^ | 2.21±0.18^b^ |  | 4.27±0.17^b^ | 9.64±0.74^b^ | 3.00±0.24^a^ |  | 7.50±0.61^b^ | 13.26±1.15^b^ | 3.61±0.44^b^ |
| BF | 4.05±0.23^a^ | 7.67±0.84^a^ | 2.50±0.35^a^ |  | 5.04±0.22^a^ | 11.17±0.97^a^ | 3.07±0.30_a_ |  | 8.46±0.55^a^ | 14.88±1.54^a^ | 4.01±0.38^a^ |
| Treatment | Time | | | | | | | | | | |
|  | 20d | | | | 25d | | | | 30d | | |
|  | Plant height/cm | Crown width/cm | Leaf width/cm |  | Plant height/cm | Crown width/cm | Leaf width/cm |  | Plant height/cm | Crown width/cm | Leaf width/cm |
| CK | 8.93±0.27^c^ | 14.64±0.96^d^ | 3.01±0.40^c^ |  | 12.52±0.44^d^ | 22.67±1.05^c^ | 4.28±0.57^b^ |  | 12.93±0.67^c^ | 22.86±1.59^b^ | 4.48±0.39^d^ |
| CF | 10.31±0.79^b^ | 17.07±0.92^c^ | 3.63±0.35^b^ |  | 13.64±0.53^c^ | 25.50±1.20^b^ | 4.28±0.27^b^ |  | 14.07±1.06^b^ | 25.64±2.44^a^ | 4.89±0.34^c^ |
| OF | 10.68±1.11^b^ | 18.83±0.64^b^ | 3.79±0.65^b^ |  | 14.27±0.42^b^ | 25.74±2.10^b^ | 4.77±0.55^b^ |  | 14.72±0.75^ab^ | 25.78±3.22^a^ | 5.37±0.26^b^ |
| BF | 12.84±0.74^a^ | 21.81±0.70^a^ | 4.70±0.70^a^ |  | 15.31±0.44^a^ | 28.13±0.84^a^ | 5.80±0.51^a^ |  | 15.52±0.93^a^ | 26.93±1.23^a^ | 6.90±0.22^a^ |

CK = untreated control; CF = chemical fertilizer; OF = organic fertilizer; BF = bioorganic fertilizer.

**Supplementary** **Table** **3.** Effects of different fertilizer treatments on *Brassica chinensis* L. plant growth.

| Treatment | Time | | | | | | | |
| --- | --- | --- | --- | --- | --- | --- | --- | --- |
|  | 5d | | | 10d | | | 15d | |
|  | Leaf number | Leaf SPAD value |  | Leaf number | Leaf SPAD value |  | Leaf number | Leaf SPAD value |
| CK | 5.78±0.58^b^ | 41.96±2.42^a^ |  | 7.67±0.47^b^ | 41.87±3.00^a^ |  | 9.72±0.63^c^ | 43.67±2.06^a^ |
| CF | 6.44±0.50^a^ | 40.52±1.38^a^ |  | 8.39±0.66^a^ | 41.76±1.64^a^ |  | 10.61±0.97^b^ | 44.12±3.06^a^ |
| OF | 6.61±0.39^a^ | 41.59±1.72^a^ |  | 8.39±0.46^a^ | 41.97±2.03^a^ |  | 10.78±0.63^b^ | 44.61±2.10^a^ |
| BF | 6.67±0.33^a^ | 40.99±1.41^a^ |  | 8.94±0.93^a^ | 43.52±1.23^a^ |  | 11.72±1.00^a^ | 45.39±2.15^a^ |
| Treatment | Time | | | | | | | |
|  | 20d | | | 25d | | | 30d | |
|  | Leaf number | Leaf SPAD value |  | Leaf number | Leaf SPAD value |  | Leaf number | Leaf SPAD value |
| CK | 12.22±1.00^c^ | 42.84±1.70^b^ |  | 13.78±1.13^b^ | 44.37±4.18^b^ |  | 17.44±0.68^c^ | 45.17±4.11^b^ |
| CF | 13.06±0.83^bc^ | 43.09±1.52^b^ |  | 14.22±0.63^b^ | 45.19±1.79^ab^ |  | 18.28±1.27^bc^ | 45.84±2.07^b^ |
| OF | 13.44±0.60^b^ | 42.54±1.79^b^ |  | 15.39±0.61^b^ | 45.97±2.30^ab^ |  | 18.50±0.53^b^ | 46.79±1.82^b^ |
| BF | 14.50±1.08^a^ | 45.22±1.78^a^ |  | 16.50±0.88^a^ | 47.91±0.82^a^ |  | 20.39±1.07^a^ | 50.03±2.07^a^ |

CK = untreated control; CF = chemical fertilizer; OF = organic fertilizer; BF = bioorganic fertilizer.

**Supplementary Table 4.** Effects of different fertilizer treatments on the physicochemical properties of soil.

| **Treatment** | **Alkaline hydrolysis nitrogen**  **(mg/kg)** | **Available P (mg/kg)** | **Available K (mg/kg)** | **Organic matter (g/kg)** | **pH** |
| --- | --- | --- | --- | --- | --- |
| CK | 93.13 ± 2.37^b^ | 127.47 ± 4.82^c^ | 132.05 ± 4.50^c^ | 33.72 ± 1.00^b^ | 6.22 ± 0.01^c^ |
| CF | 95.03 ± 1.47^ab^ | 138.55 ± 6.13^b^ | 135.33 ± 4.53^c^ | 32.95 ± 1.07^b^ | 6.15 ± 0.03^d^ |
| OF | 97.53 ± 3.23^a^ | 142.78 ± 1.50^ab^ | 195.43 ± 4.46^b^ | 40.74 ± 0.54^a^ | 6.53 ± 0.01^b^ |
| BF | 98.25 ± 2.14^a^ | 146.42 ± 7.56^a^ | 214.20 ± 2.08^a^ | 39.56 ± 1.12^a^ | 6.60 ± 0.08^a^ |

CK = untreated control; CF = chemical fertilizer; OF = organic fertilizer; BF = bioorganic fertilizer. Means (N = 6) within the same column followed by the same letter are not statistically different (P = 0.05) according to Duncan’s new Multiple-Range test.

**Supplementary Table 5.** Spearman correlations between agronomic traits and soil environmental factors.

| **Item** | **AHN** | **AP** | **AK** | **OM** | **pH** |
| --- | --- | --- | --- | --- | --- |
| Fresh weight | 0.51^*^ | 0.59^**^ | 0.70^***^ | 0.53^**^ | 0.39 |
| SPAD value | 0.28 | 0.42^*^ | 0.52^**^ | 0.31 | 0.56^**^ |
| Nitrate content | -0.35 | -0.31 | -0.72^***^ | -0.45^*^ | -0.70^***^ |
| Soluble sugar content | 0.53^**^ | 0.71^***^ | 0.91^***^ | 0.74^***^ | 0.71^***^ |
| Soluble protein content | 0.43^*^ | 0.65^***^ | 0.71^***^ | 0.4 | 0.33 |

AHN is alkaline hydrolysis nitrogen; AP is available phosphorus; AK is available potassium; OM is organic matter.* 0.01 < P ≤ 0.05; ** 0.001 < P ≤ 0.01; *** P ≤ 0.001.

**Supplementary Table 6.** Bacterial and fungal α-diversity indexes of the four treatments.

|  | Treatment | Numbers of OTUs | Chao 1 | Shannon |
| --- | --- | --- | --- | --- |
| Bacteria | CK | 2243±38^a^ | 3068.43±37.99^a^ | 5.96±0.07^a^ |
|  | CF | 2182±61^a^ | 2959.26±131.19^ab^ | 5.94±0.05^a^ |
|  | OF | 2091±46^b^ | 2840.23±69.38^b^ | 5.82±0.06^b^ |
|  | BF | 2034±106^b^ | 2867.31±158.67^b^ | 5.65±0.12^c^ |
| Fungi | CK | 594±28^a^ | 689.57±36.35^a^ | 3.78±0.15^a^ |
|  | CF | 573±40^a^ | 702.45±56.72^a^ | 3.70±0.28^a^ |
|  | OF | 486±20^b^ | 640.60±46.33^a^ | 3.24±0.14^b^ |
|  | BF | 498±51^b^ | 664.49±56.97^a^ | 3.25±0.19^b^ |

CK = untreated control; CF = chemical fertilizer; OF = organic fertilizer; BF = bioorganic fertilizer.

**Supplementary Table 7.** Topological features of the co-occurrence network of bacteria and fungi in soil.

| Treatment | Number of nodes | Number of edges | Positive correlation | Negative correlation | Average degree | Average clustering coefficient | Network Density | Average path length |
| --- | --- | --- | --- | --- | --- | --- | --- | --- |
| CK | 155 | 1168 | 51.63 | 48.37 | 12.360 | 0.491 | 0.066 | 3.093 |
| CF | 163 | 1350 | 50.75 | 49.25 | 14.286 | 0.505 | 0.076 | 3.009 |
| OF | 180 | 1537 | 56.59 | 43.41 | 16.265 | 0.475 | 0.087 | 2.901 |
| BF | 189 | 1541 | 57.82 | 42.18 | 16.307 | 0.524 | 0.087 | 2.936 |

CK = untreated control; CF = chemical fertilizer; OF = organic fertilizer; BF = bioorganic fertilizer.

## Supplementary Figure

##
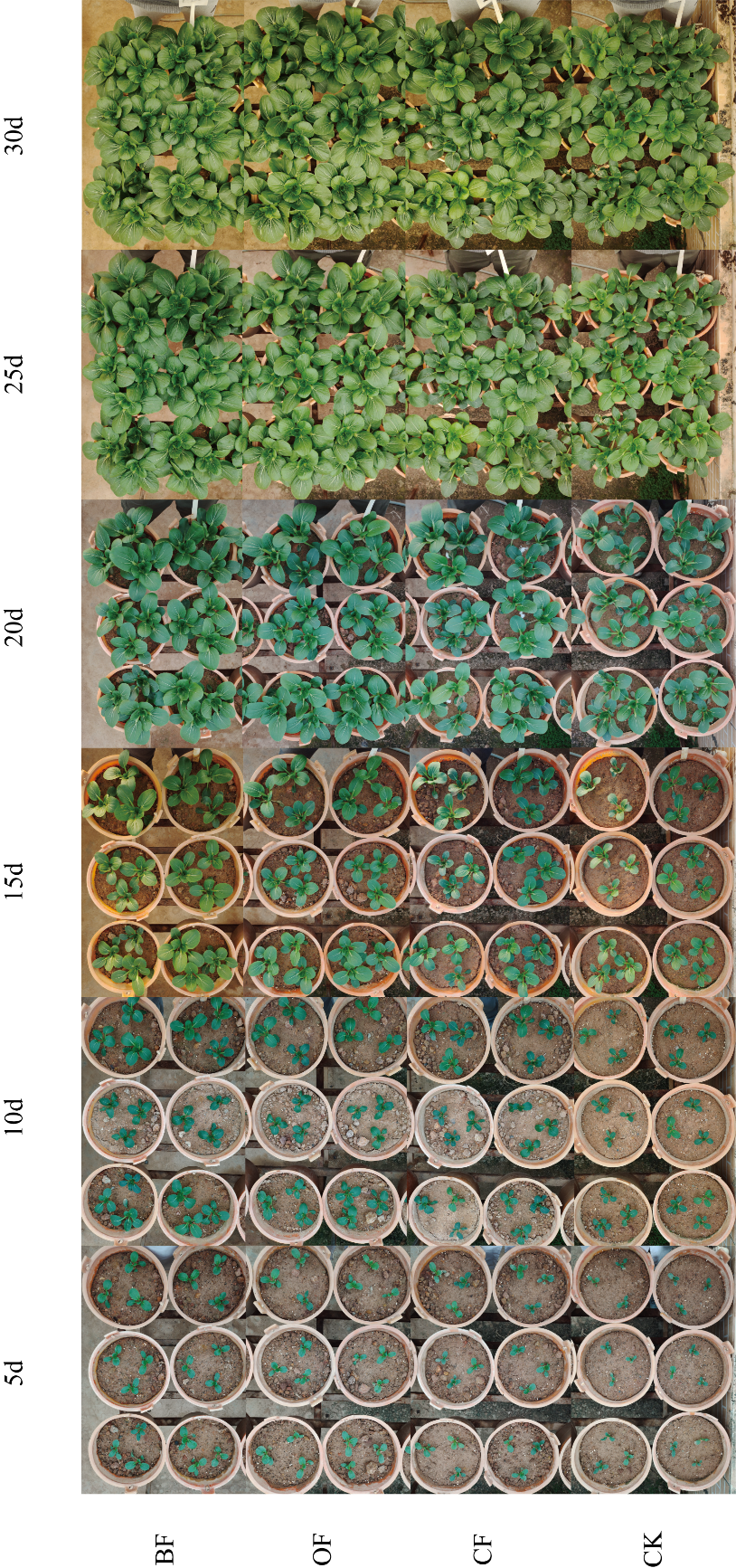


## Supplementary Figure 1. Plant seedlings grown in the pot soil field for 30 days post transplanting. CK is the untreated control; CF is chemical fertilizer; OF is organic fertilizer; and BF is bioorganic fertilizer.


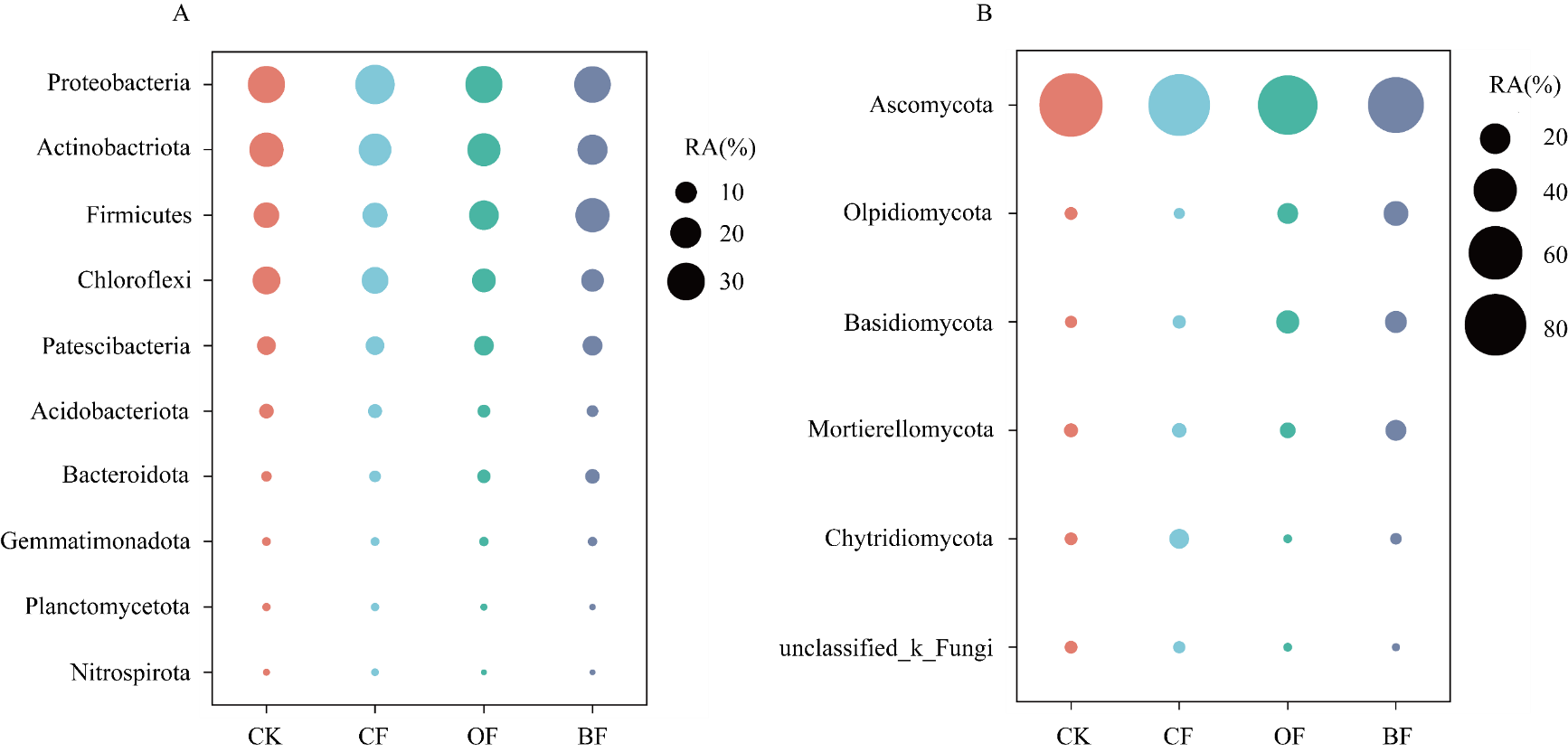


**Supplementary Figure 2.** Bubble chart of bacterial (A) and fungi (B) phylum in different fertilizer treatments. CK is the untreated control; CF is chemical fertilizer; OF is organic fertilizer; and BF is bioorganic fertilizer. Circular area represent the average relative abundance across the six replicate libraries for soil samples collected from each treatment.


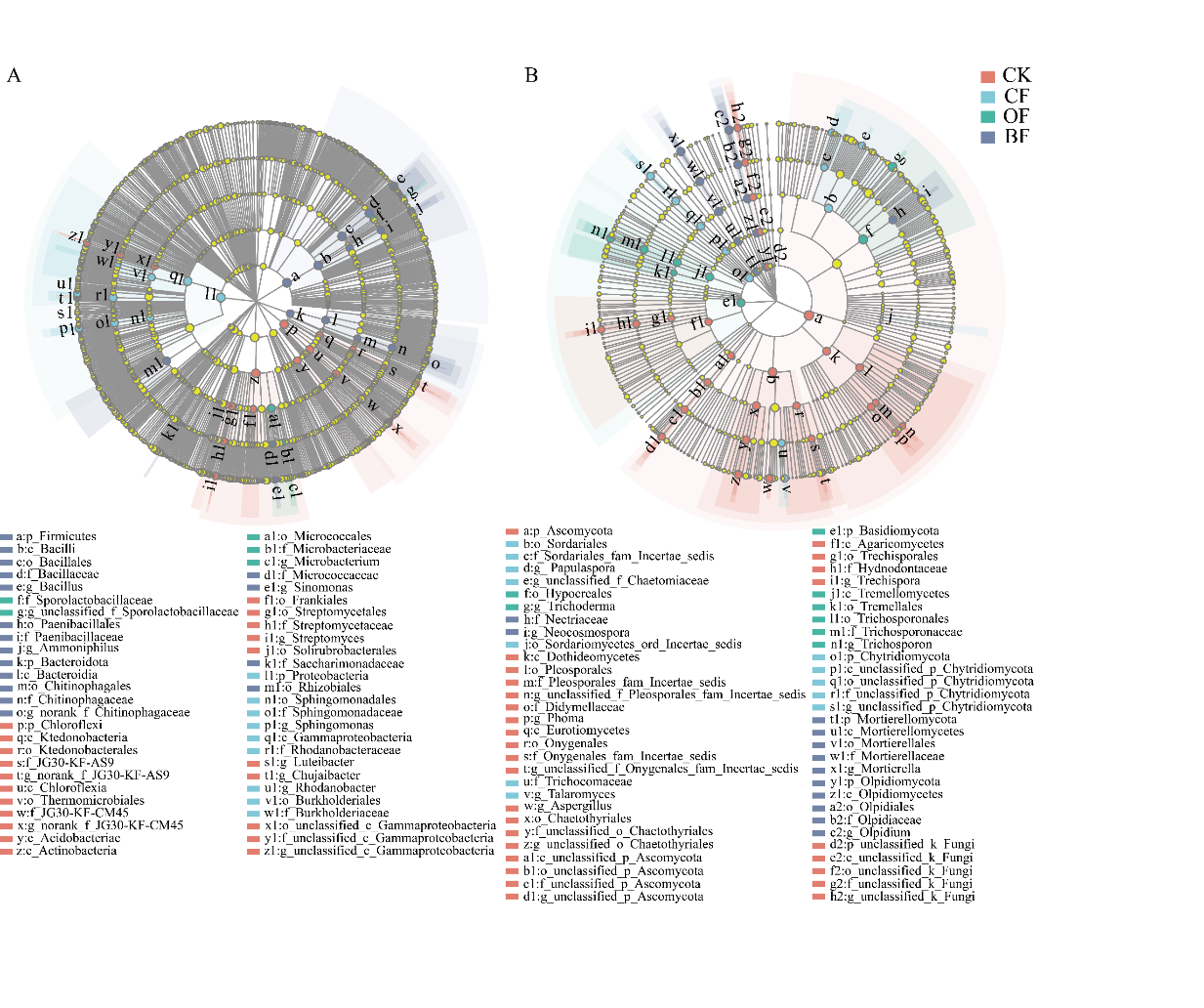


**Supplementary Figure 3.** LEfSe cladogram analysis of the differentially abundant species in soil bacterial (A, LDA = 3.5) and fungal (B, LDA = 3.5) communities in response to different fertilizer treatments (*p* = 0.05). CK is the untreated control; CF is chemical fertilizer; OF is organic fertilizer; and BF is bioorganic fertilizer. The figure shows five rings in the cladogram, from inside to outside, representing the phylum, class, order, family and genus level, respectively. The different color nodes (except yellow) on the ring represent significant changes in taxonomic composition due to the treatments.


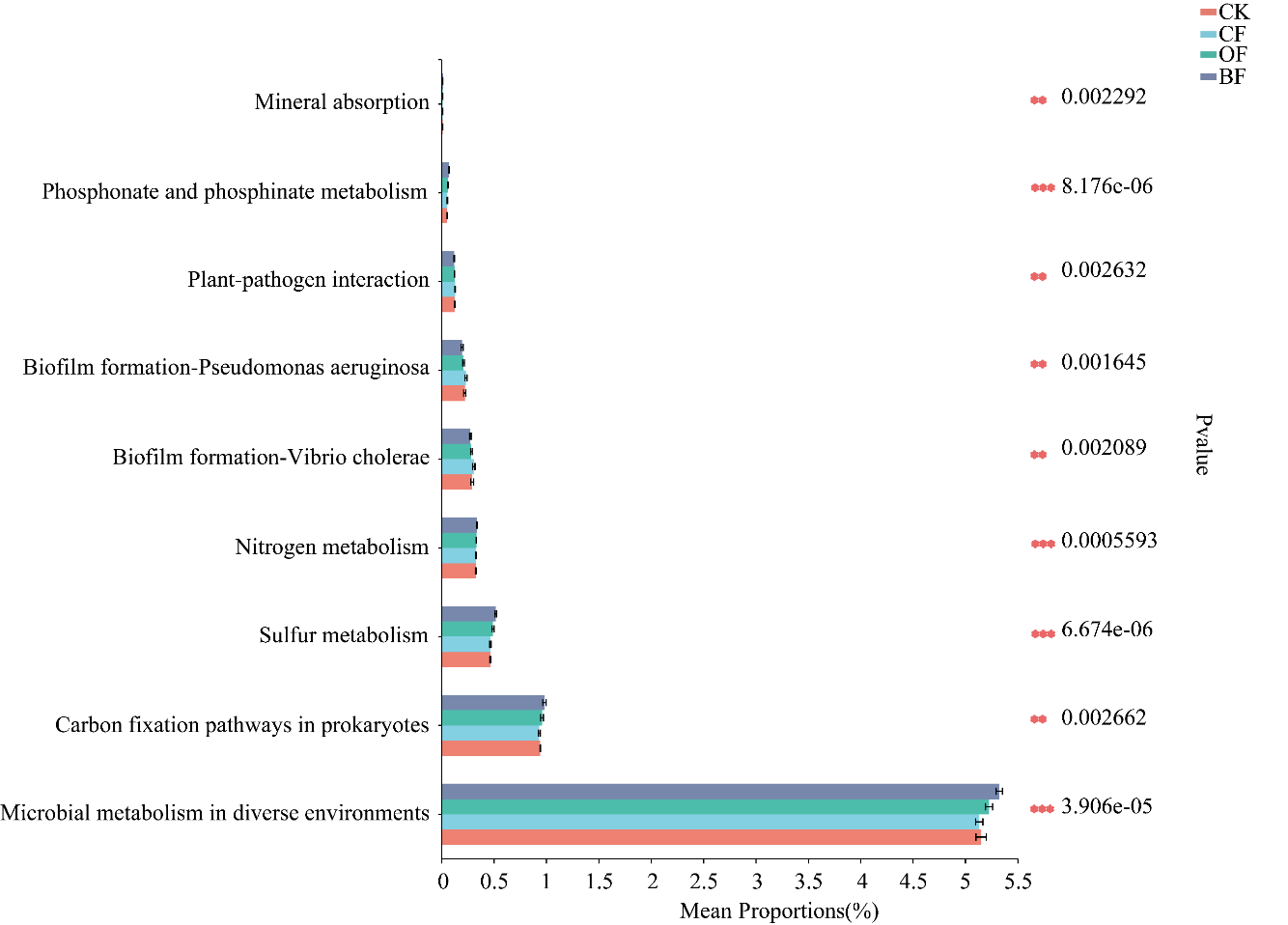


**Supplementary Figure 4.** The functional profiles of bacterial communities (KEGG level 3). CK is the untreated control; CF is chemical fertilizer; OF is organic fertilizer; and BF is bioorganic fertilizer.


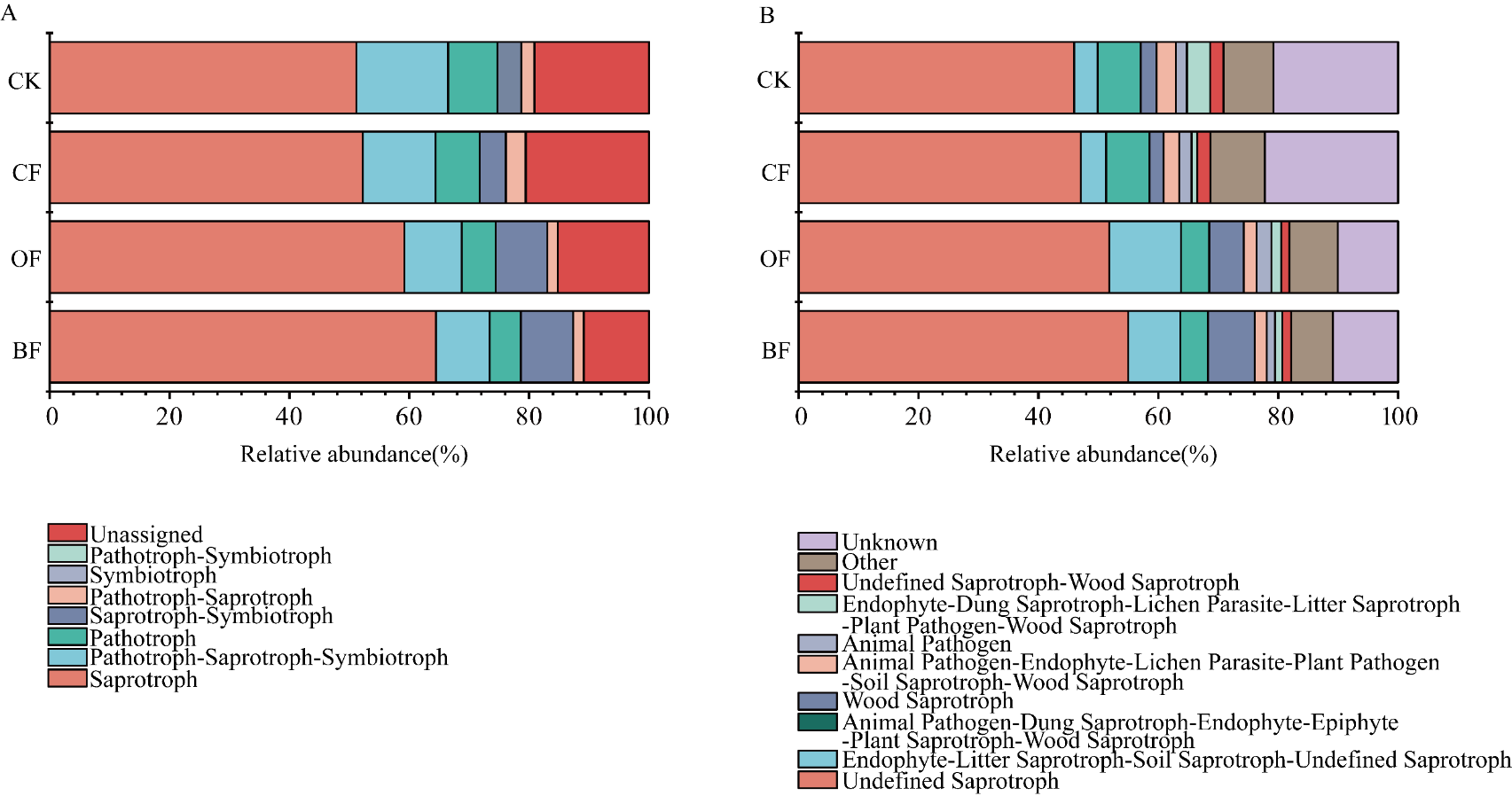


**Supplementary Figure 5.** Relative abundance of fungal trophic groups (A) and functional groups (B). CK is the untreated control; CF is chemical fertilizer; OF is organic fertilizer; and BF is bioorganic fertilizer.
