# Supplementary material for: Bioorganic fertilizer promotes pakchoi growth and shapes the soil microbial structure
Source: Front Plant Sci. 2022 Nov 8;13:1040437. doi: 10.3389/fpls.2022.1040437 (PMC9679507; doi:10.3389/fpls.2022.1040437)
Supplement: Supplementary file 2 [file DataSheet_2.docx]

**Additional file**

**Materials and Methods**

**Soil DNA extraction, PCR amplification and high-throughput gene sequencing analysis**

The instructions of DNeasy® Powersoil® DNA Isolation Kit (QIAGEN GmbH, Germany) were followed to extract total genomic DNA from each 0.25 g soil sample. The quality of the extracted DNA was checked by 1% (w/v) agarose gel electrophoresis, and the DNA concentration was determined by a NanoDrop 2000 Spectrophotometer (Thermo Fisher Scientific Inc., USA).

Two universal primers 338F (5’-ACTCCTACGGGAGCAGCAG-3’) -806R (5’-GGACTACHVGGGTWTCTAAT-3’) and ITS1F (5’-CTTGGTCATTTAGAGGAAGTAA-3’) -ITS2R (5’-GCTGCGTTCTTCATCGATGC-3’) were used to perform PCR amplification. MiSeq sequencing on the V3-V4 region of the bacterial 16SrRNA gene and the fungal ITS1 region (Schoch et al., 2012; Mori et al., 2014). The PCR amplification conditions were an initial denaturation at 95 ℃ for 3 min, followed by 28 cycles of denaturation at 95 ℃ for 30 s, annealing at 55 ℃ for 30 s, elongation at 72 ℃ for 45 s and a final extension at 72 ℃ for 10 min. PCR products were purified by E.Z.N.A.® Gel Extraction Kit (Omega, USA) and then sequenced at commercial sequencing company (Majorbio Bio-Pharm Technology Co., Ltd., Shanghai, China) with the Illumina Hiseq2500 platform.

FLASH and trimmomatic software were used to perform sequence splicing and quality control filtering on the original data obtained from MiSeq sequencing (Magoc and Salzberg, 2011). Sequences with a sequence length of 50 bp, quality less than 20, and unclear bases were removed in order to obtain high-quality, optimized sequence data for subsequent information analysis (Edgar et al., 2011). UPARSE pipeline was used to cluster the optimized sequence that were 97% similar into operable taxa (OTU). To obtain the species annotation information, we compared representative sequences of OTUs against the Silva (SSU123) 16S rRNA Database and the fungal (ITS) Unite Database using the RDP Classifier algorithm (Li et al., 2022). Sequence data associated with this project have been deposited in the NCBI Short Read Archive database (Accession Number: PRJNA773615).

**Network analysis**

Network analysis was performed based on OTUs to explore the relationships between soil microbial taxa. OTUs with a relative abundance of less than 0.1% and an occurrence frequency of less than 4 out of 6 data columns were deleted to reduce rare OTUs in the data set. Spearman correlation and network properties were calculated using the psych and igraph packages in R (version 4.2.0). After adjusting for Benjamini-Hochberg’s false discovery rate, we retained results with an absolute r value greater than 0.8 and a *P* value less than 0.05. The network was visualized using Gephi 0.9.5 (https://gephi.org/). To describe the topology of the resulting networks, a set of measures (number of nodes and edges, average path length, average degree, graph density, clustering coefficient, correlation, and modularity) was calculated using Gephi. (Benjamini and Hochberg, 1995; Li et al., 2019).

**References**

Benjamini, Y., and Hochberg, Y. (1995). Controlling the false discovery rate - a practical and powerful approach to multiple testing. *Journal of the Royal Statistical Society Series B-Statistical Methodology* 57(1)**,** 289-300. doi: 10.1111/j.2517-6161.1995.tb02031.x.

Edgar, R.C., Haas, B.J., Clemente, J.C., Quince, C., and Knight, R. (2011). UCHIME improves sensitivity and speed of chimera detection. *Bioinformatics* 27(16)**,** 2194-2200. doi: 10.1093/bioinformatics/btr381.

Li, Q., Zhang, D., Song, Z., Ren, L., Jin, X., Fang, W., et al. (2022). Organic fertilizer activates soil beneficial microorganisms to promote strawberry growth and soil health after fumigation. *Environmental Pollution* 295. doi: 10.1016/j.envpol.2021.118653.

Li, Y., Wu, H., Shen, Y., Wang, C., Wang, P., Zhang, W., et al. (2019). Statistical determination of crucial taxa indicative of pollution gradients in sediments of Lake Taihu, China. *Environmental Pollution* 246**,** 753-762. doi: 10.1016/j.envpol.2018.12.087.

Magoc, T., and Salzberg, S.L. (2011). FLASH: fast length adjustment of short reads to improve genome assemblies. *Bioinformatics* 27(21)**,** 2957-2963. doi: 10.1093/bioinformatics/btr507.

Mori, H., Maruyama, F., Kato, H., Toyoda, A., Dozono, A., Ohtsubo, Y., et al. (2014). Design and experimental application of a novel non-degenerate universal primer set that amplifies prokaryotic 16S rRNA genes with a low possibility to amplify eukaryotic rRNA genes. *DNA Research* 21(2)**,** 217-227. doi: 10.1093/dnares/dst052.

Schoch, C.L., Seifert, K.A., Huhndorf, S., Robert, V., Spouge, J.L., Levesque, C.A., et al. (2012). Nuclear ribosomal internal transcribed spacer (ITS) region as a universal DNA barcode marker for Fungi. *Proceedings of the National Academy of Sciences of the United States of America* 109(16)**,** 6241-6246. doi: 10.1073/pnas.1117018109.
